# Prevalence of Depression Among People Living with HIV on Antiretroviral Therapy in Africa: A Systematic Review and Meta-Analysis

**DOI:** 10.3390/healthcare13010085

**Published:** 2025-01-06

**Authors:** Dimakatso M. Molapo, Kabelo Mokgalaboni, Wendy N. Phoswa

**Affiliations:** Department of Life and Consumer Sciences, College of Agriculture and Environmental Sciences, University of South Africa (UNISA), Science Campus, Private Bag X6, Florida, Roodepoort 1710, South Africa; 21660107@mylife.unisa.ac.za (D.M.M.); mokgak@unisa.ac.za (K.M.)

**Keywords:** antiretroviral therapy, human immune deficiency virus, depression

## Abstract

Background: HIV is a global health issue, with the highest number of infected individuals found in sub-Saharan Africa. The coexistence of HIV with depression is a huge challenge. This study aimed to investigate the prevalence of depression in people living with HIV (PLWHIV) who are on antiretroviral therapy (ART) in Africa. Method: PubMed, Scopus, and bibliographic screening were used to identify suitable literature. The study adhered to guidelines outlined by Preferred Reporting Items for Systematic Reviews and Meta-Analyses. The Newcastle–Ottawa guideline was used to assess the quality of the included cross-sectional studies. Subgroup analysis and meta-regression were subsequently conducted following the meta-analyses, based on heterogeneity. A meta-analysis software online tool and Jamovi software (version 2.4.8.0) were used to analyse the data, and the results were presented as prevalence and 95% confidence intervals. Results: Thirty-four cross-sectional studies identified from the databases were deemed relevant. The overall sample size was 21,143 PLWHIV on ART in African countries. The analysed data showed the prevalence of depression to be 36%, with 95% CI (27% to 40%), *p* < 0.01, in Africa. However, the subgroup showed that the highest prevalence was in Northern Africa, with a prevalence of 41% with 95% CI (20% to 50%), *p* < 0.01, followed by those in Southern and Eastern Africa, with a prevalence of 38% with 95% CI (27% to 49%) and 39% with 95% CI (26% to 50%), *p* < 0.01, respectively. The lowest prevalence was observed in Western Africa, with a prevalence of 20% with 95% CI (14% to 27%), *p* < 0.01. Conclusions: Our findings show that there is a higher prevalence of depression among PLWHIV who are on ART in Africa. It is crucial to correctly recognise and provide proper care for depression to optimise HIV treatment and enhance treatment adherence in this population.

## 1. Introduction

Human immunodeficiency virus (HIV) is a worldwide health challenge, and most individuals who are infected are situated in sub-Saharan Africa [1]. In South Africa, the prevalence of HIV has risen exponentially, and it was reportedly 12.7% in 2024, which translates to 8 million people [2]. HIV attacks the immune system by targeting the T helper cells, namely the CD4^+^ helper cells [3]. The loss of CD4^+^ T helper cell function causes the host’s immune system to become weakened and prone to infections and diseases [3]. When HIV is not treated, it leads to acquired immunodeficiency syndrome (AIDS) [4]. HIV affects people of all ages and all genders. However, a significant number of women are affected compared to men [1].

Antiretroviral therapy (ART) is recommended by the World Health Organisation (WHO) as a main treatment to prevent the spread of HIV, as currently, there is no definite cure for HIV except for stem cell transplantation techniques that have been employed in rare cases [4,5]. Infected pregnant women are prescribed ART to prevent HIV transmission to the unborn baby [4]. The availability of ART has transformed HIV into a chronic disease which can be managed, improving the overall quality of life [6]. Additionally, pre-exposure prophylaxis (PrEP) and post-exposure prophylaxis (PEP) are widely used to prevent HIV transmission, especially in those at high risk of infections [4]. The coexistence of HIV with mental health disorders is a huge challenge [6]. It has been noted that more than 50% of people living with HIV (PLWHIV) are suffering from depression, mood disorder, anxiety disorder, sleep disorder, and somatic complaints [7]. Post-traumatic stress disorder (PTSD) has also been reported in PLWHIV [8,9]. It has been reported that the number of HIV-infected females who experience depression, anxiety, and PTSD is higher than that of HIV-infected males [10]. Additionally, women living with HIV have been diagnosed with depression at a 45% higher rate than women who are not living with HIV [10]. Depression has been reported in ART-treated individuals [7,11]. Some studies suggest that poor ART adherence promotes various mental health disorders [7,11,12]. It is, however, not clear whether ART independently induces depression, or whether this is dependent on the HIV status of the patient. Previous studies have reported that depression and anxiety are associated with poor ART adherence, suboptimal retention in HIV care, or inferior HIV-related outcomes [11,13]. It is also suggested that HIV can promote mental health and substance use due to the stigma associated with the condition [13,14]. The stigma not only affects self-esteem and social relationships but also has implications for the control and management of HIV [14]. For instance, some PLWHIV tend not to collect their ART medication in public health centres due to the stigma associated with the condition; some do not adhere to medication due to failure to disclose to those close to them, and this could lead to depression. Individuals suffering from depression and HIV are more likely to abuse substances, which may lead to poor adherence, increase HIV transmission, and exacerbate HIV complications, which can result in the development of AIDS [7]. While the association between HIV, ART, and depression has been reported in longitudinal studies [15], the prevalence of such has not been properly documented, especially in Africa, where the prevalence of HIV is substantial. Therefore, the current study aims to investigate the prevalence of depression in PLWHIV on ART in Africa.

## 2. Methods

### 2.1. Search Strategy and Literature Search

This study comprised a systematic review and meta-analysis of published studies. The study adhered to guidelines outlined by Preferred Reporting Items for Systematic Reviews and Meta-Analyses (PRISMA) [16]. The online databases, PubMed and Scopus, were searched comprehensively for eligible literature. A manual search was also performed to identify the relevant literature. The following Medical Subject Heading (MeSH) terms and keywords were used to search the databases: “HIV” OR “Human Immunodeficiency Virus” AND “AIDS” OR “Acquired immune deficiency syndrome” AND “ART” OR “Antiretroviral therapy” AND “Depression”. This study is reported in accordance with PRISMA (Supplementary File S1).

### 2.2. Eligibility Criteria (Inclusion and Exclusion Criteria) and Study Selection

The eligibility of the studies was determined by the Population, Intervention, Comparison, Outcome, Study Design (PICOS) framework, as illustrated in Table 1.

The studies found were exported to the citation manager Mendeley Reference Manager (version 2.125.2). This software was used to remove duplicate records. The titles and abstracts of articles that remained after the exclusion of duplicates were assessed for eligibility, according to the inclusion and exclusion criteria. The full text of all eligible studies was reviewed by two independent reviewers (D.M.M. and W.N.P.). The disagreements between the reviewers regarding the eligible studies included were settled by a third reviewer (K.M.). The reference list of eligible studies and reviews was assessed for the determination of more eligible studies. Additionally, the studies included were conducted in Africa and published in English. The study design included cross-sectional studies amongst PLWHIV who were on ART. Studies that did not show the outcomes of interest, conducted in children, adolescents, and pregnant females were excluded.

### 2.3. Data Extraction, Data Synthesis, Quality Assessment, and Data Analysis

A data extraction form was used by the two reviewers (D.M.M. and W.N.P.) to extract data from the studies. The data extracted in each study included author, date or year of publication, study title, study design, age, gender of the PLWHIV, sample size, country, and ART. The third reviewer (K.M.) assisted the reviewers (D.M. and W.P.) in reaching a consensus regarding any disagreements. The data were analysed using an online meta-analysis tool, i.e., https://metaanalysisonline.com/ (accessed on 5 November 2024), and Jamovi software (version 2.4.8.0). The number of PLWHIV that developed depression and the sample size were extracted in each cross-sectional study. Data were reported as prevalence and 95% confidence intervals. Meta-regression and subgroup analysis were conducted to investigate the source of heterogeneity. A funnel plot was graphically used to illustrate the presence or absence of publication bias. In contrast, the Egger regression test was used to statistically substantiate bias.

### 2.4. Risk the Quality Assessment

The quality of each cross-sectional study was assessed using the Newcastle–Ottawa assessment scale (NOS) [17]. The scale considers three main domains: selection, comparability, and outcomes. Two independent researchers (D.M.M. and W.N.P.) made the overall judgment. In the event of disagreement, a third researcher (K.M.) determined the domain in question. The final decision was based on a rating system in which studies that scored seven and above were considered to be of high quality, those between four and six were deemed of moderate quality, and any score lower than four was regarded as low quality.

## 3. Results

### 3.1. General Overview of Search and Selection

**Figure 1:**
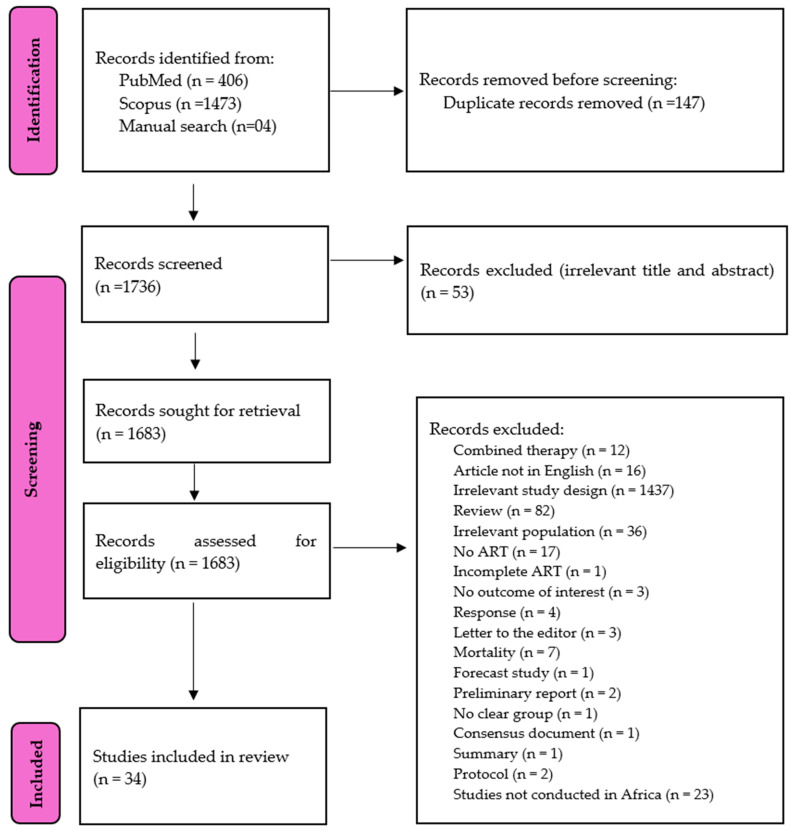
Preferred Reporting Items for Systematic Review and Meta-Analyses (PRISMA) flow diagram indicating the review process.

The search from PubMed and Scopus, along with a manual search of references, yielded 1883 records. The records were first screened using the Mendeley Reference Manager (version 2.125.2), and 147 were found to be duplicates. Secondly, we screened the remaining records by assessing the title, abstract, and keywords, and 54 were excluded. The remaining 1683 articles were evaluated, and 1649 were determined to be irrelevant and were further excluded. They were excluded for reasons such as irrelevant outcomes, articles not published in English, studies without ART or incomplete ART, correspondences, those without a clear group, those limited by mortality, and a population that is not relevant (pregnant females, adolescents, and children), as shown in Figure 1. The total number of cross-sectional studies included was 34, with 21,178 PLWHIV, all of whom were on ART [18,19,20,21,22,23,24,25,26,27,28,29,30,31,32,33,34,35,36,37,38,39,40,41,42,43,44,45,46,47,48,49,50,51] (Figure 1).

### 3.2. Characteristics of Included Studies

The characteristics of the included studies are summarised in Table 2. These studies were performed from 2013 to 2024, with sample sizes ranging from 103 to 8675. These studies [18,19,20,21,22,23,24,25,26,27,28,29,30,31,32,33,34,35,36,37,38,39,40,41,42,43,44,45,46,47,48,49,50,51] were all conducted in Africa, specifically, two studies from Cameroon [35,43], one study from Cote D’Ivoire and Senegal [37], thirteen studies from Ethiopia [21,22,24,25,28,30,33,34,36,41,45,48,50], one study from the Republic of Guinea [47], one study from Kenya [49], one study from Malawi [23], one study from Mozambique [19], two studies from Nigeria [26,40], one study from Somalia [44], seven studies from South Africa [18,27,32,38,42,46,51], one study from Tanzania [29], and three studies from Uganda [20,31,39]. The study design type for all included studies was cross-sectional. While not all studies reported age, the overall mean age and SD for those reporting age was 38.4 ± 11 years. There were six classes of methods of measuring depression. The following methods were used to measure depression in these studies: class 1, nineteen studies used the Patient Health Questionnaire (PHQ-9) [21,23,24,25,26,28,29,30,33,34,35,36,40,43,44,45,49,50,51]; class 2, five studies used the Centre for Epidemiological Studies Depression Scale (CES-D) [18,37,41,42,46]; class 3, three studies used the Hospital Anxiety and Depression Scale (HADS) questionnaire [22,47,48]; class 4, two studies used the Beck Depression Inventory scale [32,39]; class 5, one study used the General Health Questionnaire (GHQ12) [27]; and class 6, which was subgrouped into “other”, as the five studies used different methods (an adherence survey questionnaire that included a depressive symptoms score, the Likert method, the Hopkins Symptom Checklist for depression (HSCL), a structured diagnostic interview, the Brazilian version of the Portuguese language Mini International Neuropsychiatric Interview (MINI) Plus 4.0.0, and the Zung Self-Rating Depression Scale) [19,20,27,31,38].

### 3.3. Quality of Included Trials

All the cross-sectional studies that were included scored between 6 and 8 stars in terms of quality, as shown in Supplementary File S2, Table S1. Of the 36 (64%) included studies, 23 scored between 7 and 8 and were classified as high-quality studies. Only 13 (36%) studies scored 6 stars and were classified as moderate-quality studies.

### 3.4. Prevalence of Depression in PLWHIV Who Are on ART in Africa

**Figure 2:**
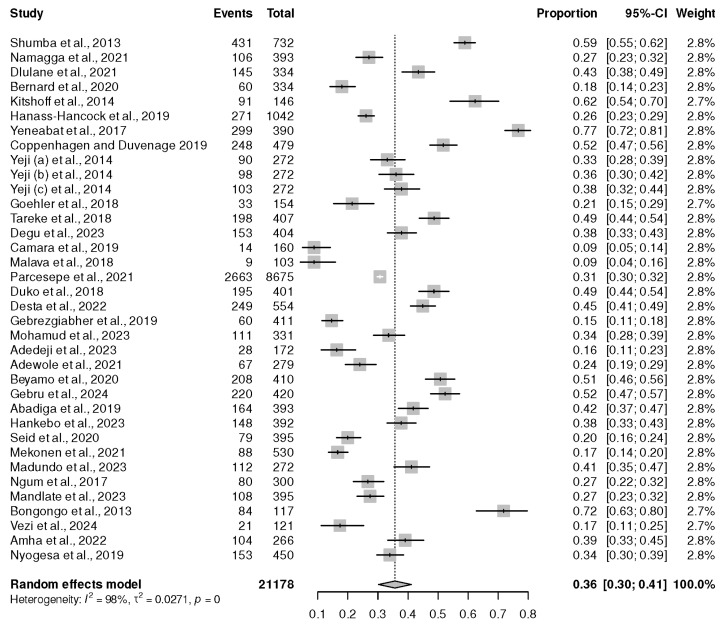
Forest plot for the prevalence of depression within the African continent. Shumba et al., 2013 [31], Namagga et al., 2021 [39], Dlulane et al., 2021 [32], Bernard et al., 2020 [37], Kitshoff et al., 2014 [42], Hanass-Hancock et al., 2019 [46], Yeneabat et al., 2017 [41], Coppenhagen and Duvenage 2019 [18], Yeji (a) et al., 2014 [27], Yeji (b) et al., 2014 [27], Yeji (c) et al., 2014 [27], Goehler et al., 2018 [20], Tareke et al., 2018 [48], Degu et al., 2023 [22], Camara et al., 2019 [47], Malava et al., 2018 [23], Parcesepe et al., 2021 [35], Duko et al., 2018 [34], Desta et al., 2022 [25], Gebrezgiabher et al., 2019 [45], Mohamud et al., 2023 [44], Adedeji et al., 2023 [26], Adewole et al., 2021 [40], Beyamo et al., 2020 [30], Gebru et al., 2024 [21], Abadiga et al., 2019 [33], Hankebo et al., 2023 [28], Seid et al., 2020 [36], Mekonen et al., 2021 [24], Madundo et al., 2023 [29], Ngum et al., 2017 [43], Mandlate et al., 2023 [19], Bongongo et al., 2013 [38], Vezi et al., 2024 [51], Amha et al., 2022 [50], and Nyogesa et al., 2029 [49].

Thirty-four cross-sectional studies, with a sample size of 21,178, found that PLWHIV on ART displayed an increased risk of depression [18,19,20,21,22,23,24,25,26,27,28,29,30,31,32,33,34,35,36,37,38,39,40,41,42,43,44,45,46,47,48,49,50,51]. The lowest prevalence was 9%, and the highest prevalence was 77% [23,41]. However, the overall prevalence from the random meta-analysis was 36% (95% CI (30%, 41%)), *p* < 0.01. The findings revealed a high level of heterogeneity (*I*^2^ of 98%), as shown in Figure 2.

#### 3.4.1. Subgroup Analysis Based on Region in the African Continent

The African continent is divided into the following four regions, as per Table 2: Southern, Western, Northern, and Eastern Africa. The subgrouping based on regions did not yield any significant changes in heterogeneity, suggesting that the regions of publication were not a significant contributor to high variation.

#### 3.4.2. Depression in Southern Africa Among PLWHIV on ART

**Figure 3:**
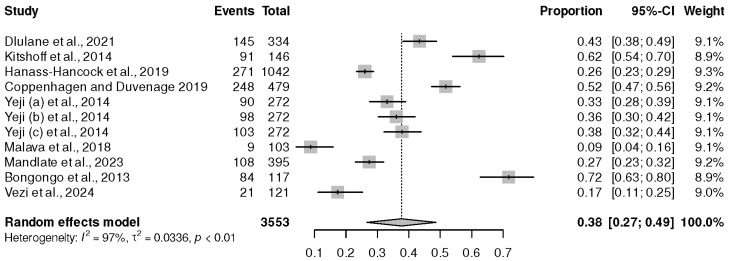
Prevalence of depression in Southern African regions. Dlulane et al., 2021 [32], Bernard et al., 2020 [37], Kitshoff et al., 2014 [42], Hanass-Hancock et al., 2019 [46], Yeneabat et al., 2017 [41], Coppenhagen and Duvenage, 2019 [18], Yeji (a) et al., 2014 [27], Yeji (b) et al., 2014 [27], Yeji (c) et al., 2014 [27], Malava et al., 2018 [23], Mandlate et al., 2023 [19], Bongongo et al., 2013 [38], and Vezi et al., 2024 [51].

Nine studies [18,19,23,27,32,38,42,46,51], with eleven arms, were conducted in Southern Africa. These cross-sectional studies had a sample size of 3553 PLWHIV on ART, and they showed a prevalence of 38% (95% CI (27%, 49%), *p* ˂ 0.01) and I^2^ = 97%, as shown in Figure 3.

#### 3.4.3. Depression in Western Africa Among PLWHIV on ART

**Figure 4:**
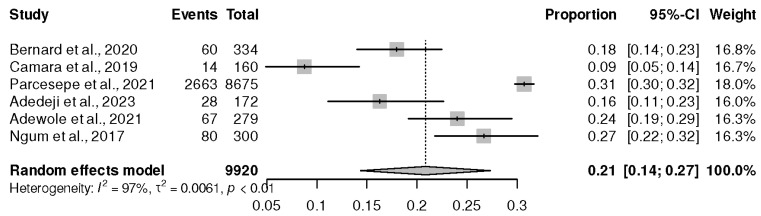
Prevalence of depression in Western African regions. Bernard et al., 2020 [37], Camara et al., 2019 [47], Parcesepe et al., 2021 [35], Adedeji et al., 2023 [26], Adewole et al., 2021 [40], and Ngum et al., 2017 [43].

Only six cross-sectional studies conducted in Western Africa [26,35,37,40,43,47] explored the risk of depression among 9920 PLWHIV on ART. The result of the random effect meta-analysis reported the prevalence of depression at 21% (95% CI (14%, 27%), *p* ˂ 0.01), and I^2^ = 97%, as shown in Figure 4.

#### 3.4.4. Depression in Northern Africa Among PLWHIV on ART

**Figure 5:**
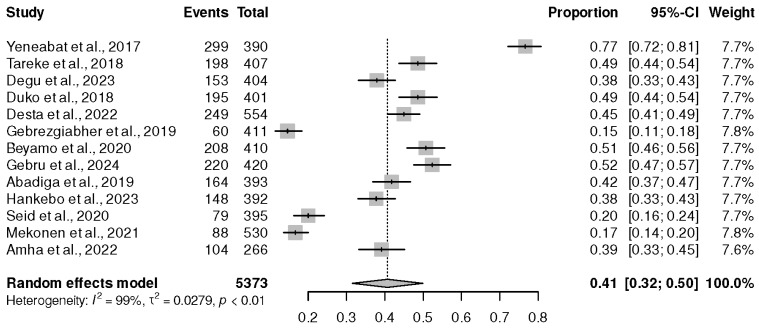
Prevalence of depression in studies published in Northern Africa. Yeneabat et al., 2017 [41], Tareke et al., 2018 [48], Degu et al., 2023 [22], Duko et al., 2018 [34], Desta et al., 2022 [25], Gebrezgiabher et al., 2019 [45], Beyamo et al., 2020 [30], Gebru [21], Abadiga et al., 2019 [33], Hankebo et al., 2023 [28], Seid et al., 2020 [36], Mekonen et al., 2021 [24], and Amha et al., 2022 [50].

Of the 34 included studies, only 13 cross-sectional studies were conducted in Northern Africa [21,22,24,25,28,30,33,34,36,41,45,48,50]. These studies were composed of 5373 PLWHIV on ART. Of concern was the highest significant prevalence of depression at 41% (95% CI (32%, 50%), *p* ˂ 0.01), and I^2^ = 99%, as shown in Figure 5.

#### 3.4.5. Depression in Eastern Africa Among PLWHIV on ART

**Figure 6:**
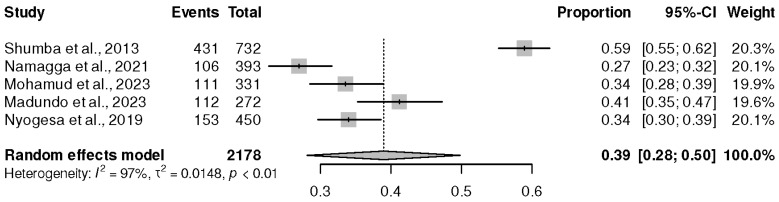
Prevalence of depression in the Eastern part of Africa. Shumba et al., 2013 [31], Namagga et al., 2021 [39], Mohamud et al., 2023 [44], Madundo et al., 2023 [29], and Nyogesa et al., 2029 [49].

The last region in Africa, in this case, Eastern Africa, had only six studies [21,30,32,40,45,50], with the lowest sample size (2332 PLWHIV on ART). However, the results from the meta-analysis revealed a prevalence of depression at 39% (95% CI (28%, 50%), *p* ˂ 0.01), and I^2^ = 97%, as shown in Figure 6.

#### 3.4.6. Subgroup Based on the Methods of Measuring Depression

Different methods used to measure depression were categorised by numbers. The summarised results are presented in Supplementary File S2, Figure S1. Briefly, there were no significant changes in the level of heterogeneity post-subgroup, based on different methods of measuring depression, suggesting that this factor did not contribute to the observed heterogeneity.

#### 3.4.7. Association Between Depression and Moderators in PLWHIV on ART

Due to the high heterogeneity found in the meta-analysis, we conducted meta-regression to investigate the association between the methods used to measure depression, the region of publication, and the prevalence of depression. According to the findings in Table 3, there is no significant association between the prevalence of depression and African regions in PLWHIV on ART (*p* = 0.655). Additionally, there was no significant association between the methods used for measuring depression and the prevalence of depression (*p* = 0.250), as shown in Table 3.

#### 3.4.8. Publication Bias

**Figure 7:**
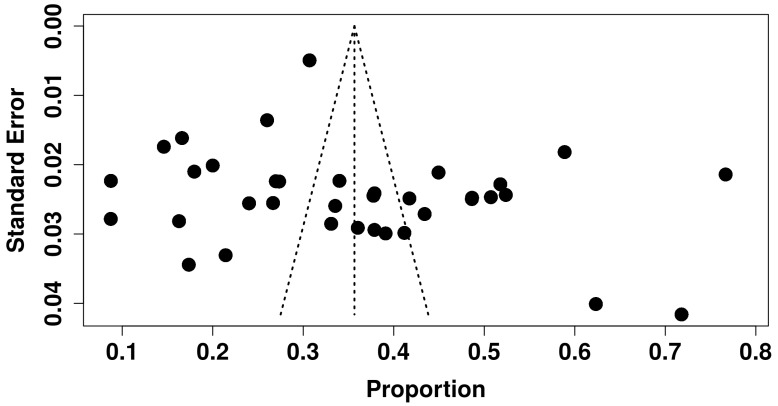
Funnel plot for the prevalence of depression in Africa.

The findings from our analysis to evaluate publication bias are displayed in the funnel plot shown in Figure 7. The distribution of studies in the plot is symmetrical, indicating no publication bias in this systematic review and meta-analysis. Additionally, the regression test (*p* = 0.021) and rank correlation test (*p* = 0.260) supported the absence of publication bias.

## 4. Discussion

Depression is a widespread mental condition that affects individuals regardless of age, gender, or race [32]. It is a major public health concern and a significant factor in the global burden of disease that affects all communities [18,32,41,42]. ART coverage has improved substantially in African countries with high HIV prevalence, with a 47.6% increase from 2015 to 2020. Nigeria and Tanzania exhibit the highest improvement, with a 72% and 71% increase in ART coverage, respectively, with the lowest coverage of 30% in Ethiopia [53]. Depression affects ART adherence, with depressive individuals less likely to use ART [54,55]. According to Nyongesa et al., 2019 [49], and other reports, depression is regarded as a common mental health disorder, and it is expected that by 2030, it will become a prominent factor in the global disease burden [29,35,36,38,43]. Depression can even be worse among PLWHIV. The results of this systemic review and meta-analysis showed that the prevalence of depression sits at 36% among PLWHIV who are on ART in Africa. The highest prevalence was observed in Northern Africa, at 41%, and the lowest was in Western Africa, at 21%. HIV and depression often result in a negative impact on both the mind and body, leading to psychiatric disorders [32,37]. The prevalence of depression is three times greater in PLWHIV than in the general population [43,49,51]. The psychological burden of HIV, along with the overall impact of chronic illness, contributes to this, making depression the most common psychiatric disorder among PLWHIV [32,33,37,38]. The previous reports indicate that individuals who were diagnosed with HIV and then seen within one month experienced the most significant levels of depression [29]. This high prevalence of depression in the early stages of diagnosis is often marked by a significant number of PLWHIV struggling to come to terms with their health status, facing ongoing discrimination, managing the demands of medical treatment, and worrying about their health and their loved ones [48]. Depression is the most widespread in the region of Northern Africa, with a prevalence of 41%. This may be attributed to North African regions having the lowest ART coverage, at 11%, in Africa, which may subject patients to stress and poor health [56]. In 2017, the prevalence of depression was significantly high in Ethiopia, with a rate of 77% [41]. This could have been motivated by various factors, including socioeconomic factors, food insecurity, poor social support, stigma, inability to disclose their status, disease among rural dwellers with inadequate access to health services, and advanced stages of AIDS [57]. Furthermore, poor adherence to ART, as well as the presence of opportunistic infections and adverse ART reactions, increased the risk of depression in this population [58]. However, more recently, in 2019, the prevalence seemed to be lower in the same country [45]. These interesting results are due to improved access to ART and enhanced mental health support [28,57,58]. Also, improved awareness about HIV may have reduced stigma, resulting in more PLWHIV seeking help and adhering to the prescribed treatment. Employment status also seems to contribute to depression, with employed PLWHIV being less likely to exhibit depression when compared to their unemployed counterparts [21,41]. Southern Africa and Eastern Africa both display a high prevalence of depression in PLWHIV, at 36%. The leading country for prevalence in the Southern region is South Africa, at 72%, while the lowest prevalence is observed in Malawi, at 9%. South Africa’s high depression rate may be linked to several factors, including its significantly high HIV infection rates, high unemployment levels, and high rates of violence, as well as crime, which are likely contributing to the depression challenges faced by the PLWHIV [32,38,51,59,60]. The lack of access to health care in South African rural areas also contributes to the prevalence of depression among PLWHIV [32]. The reason behind Malawi’s lower prevalence is attributed to the introduction of programs at clinics, established by the country’s Ministry of Health, which include the screening of depression and the provision of depression treatment for PLWHIV who are initiating ART [61]. In the Eastern African region, Uganda reported a prevalence percentage of 59% in 2013. The significant levels of depression in Uganda could have been attributed to the fact that the majority of PLWHIV were living in rural areas, had low socio-economic status, and lacked access to resources such as adequate healthcare workers and HIV care that includes mental healthcare services [31]. Most recently, Tanzania reported a prevalence of 41%, which is lower than what was previously reported in Uganda [29]. The decline in prevalence may be due to the decrease in new HIV infections in the Eastern African region [52]. Reduced new HIV infections were linked to decreases in sexual partners and higher condom usage [52,62]. In recent times, HIV incidence in Eastern Africa has been further diminished by voluntary medical male circumcision and antiviral treatment [52,62]. These recent reductions were primarily due to the expansion of antiviral therapy [52,62].

In Western Africa, the highest prevalence of depression among PLWHIV is reported in Cameroon, at 31%, and the lowest in the Republic of Guinea, at 9%. The increase in civil unrest in Cameroon has caused more people to have less access to healthcare facilities and systems, which led to a high prevalence of diseases [63]. The prevalence in this region seems to be lower than that in other regions, primarily due to improved ART coverage, as reported in Nigeria [53]. This could be made possible by the availability of ART in secondary healthcare centres, the initiation of community-based ART programs, and increased HIV and ART awareness [64,65]. The highest prevalence in the Eastern African region is likely due to the delayed initiation of ART among PLWHIV, psychological distress from chronic stress due to unemployment, and the burden of care for the disease [43,47]. Nyogesa et al., 2019, reported that participants with moderate to severe chronic pain showed higher levels of depressive symptoms relative to those with no chronic pain [49]. Inaccessible healthcare has been found to contribute to the prevalence of depression [49]. Some studies confirm that HIV-related stigma, poor ART adherence, and poor social support are significantly associated with greater symptoms of depression [28]. The build-up of worry over potential health issues can increase and appear as signs of depression [51]. Research has consistently found that the level of lack of social support, CD4 cell count, and viral load are important factors linked to depressive symptoms in PLWHIV [21,34]. It has been observed that improved social support is associated with lower levels of depression in adults receiving ART treatment. Other studies have also shown that higher levels of support from family and employment contribute to lower depression and improved quality of life for adults on ART [21,41]. While the pathogenesis of depression in PLWHIV is not fully documented, Xie et al., 2021, reported that HIV infection could be the contributing factor due to its association with inflammation [11]. HIV promotes neuroinflammation, which then impairs neurotransmitters, thus contributing to mood disorders [11]. Other researchers reported that PLWHIV with a viral load of 1000 copies/mL were nearly 2.2 times more prone to depression in comparison to individuals with lower viral loads [21,22,34]. Similarly, PLWHIV with a CD4 cell count of less than 200 cells/mm^3^ display a higher risk of depression do patients with a CD4 count greater than 350 cells/mm^3^ [21,22,41]. When it comes to related factors, females showed a 3.5 times greater risk of developing depression compared to males [22]. Females reported more depression symptoms as opposed to males; this could be because women are more likely to talk about their life experiences as failures and are more likely to be exposed to long-lasting trauma, such as sexual, physical, and emotional abuse, both during their early years and later in life [22,37]. The increased likelihood of depression in females as compared to males may also be attributed to biological or hormonal differences between the two genders [28]. The findings based on gender suggest that women may prioritise food security and body weight more than men do, and the absence of these may lead to feelings of depression [46]. It has been reported that new ART users showed a significant increase in depressive symptoms compared to those who had been using ART for a more extended period [21,66]. Depression can result in non-adherence to ART, further exacerbating depressive symptoms [67,68]. PLWHIV with a previous experience of adverse side effects from ART were found to have a higher prevalence of depression when compared to those without such a history [34,46]. Ethiopia and South Africa exhibit extremely high prevalence rates of 72% and 77%, respectively [38,41]. The high levels of prevalence in Ethiopia are closely linked with certain factors, including being over 40 years of age, feeling stigmatised or discriminated against, not adhering well to medication, and having limited support [28,30,34,48,50]. Additionally, the records found a link between depression and several factors, including opportunistic infection, adverse drug reactions, and the presence of other chronic diseases [25,33,36,45]. The high prevalence levels in South Africa are reported in the North West and Gauteng provinces. The high percentages are likely due to increased unemployment rates [38]. The level of education is also a factor; high scores for depression correlated positively with having less education [42,51]. Efforts are needed to provide support to PLWHIV experiencing depression, such as financial and moral support to boost their immune systems. The lack of support for ART patients in other African countries leads to untold suffering and additional health complications.

## 5. Research Study Limitations and Strengths

Undertaking any research study comes with limitations and constraints, which can significantly impact the results. In this research study, a minor limitation was the lack of relevant information about the exact form of ART and the number of males compared to female patients who developed depression in individual studies. Some studies did not include information about the age of participants, and there were variations in the methods used to assess depression, potentially introducing some bias. The high heterogeneity was not verified through subgroup analysis and meta-regression. One strength is that independent researchers conducted the data search using PubMed and Scopus databases to prevent bias. Another strength is our ability to identify an individual prevalence in the African regions, something that has not been done before, according to our knowledge. The quality of gathered evidence from these cross-sectional studies was classified as high (64%) and moderate (36%), based on the Newcastle–Ottawa checklist.

## 6. Conclusions and Recommendations

The findings found a prevalence of depression among PLWHIV on ART in Africa. The highest percentage was noted in the Northern region, followed by the Eastern and Southern regions, and the lowest was identified in the Western region. Proper identification and care for depression are essential for making the most of HIV treatment. This not only helps to prevent the spread of HIV but also improves the overall well-being of this population. We recommend that PLWHIV who cannot visit public clinics consider using the delivery services offered by private pharmacies, especially in the early stages of diagnosis. PLWHIV can request that their healthcare providers send their prescriptions directly to a local pharmacy, guaranteeing ongoing medication supply and delivery of their ART. In this way, PLWHIV can avoid crowded places and long queues.

## Figures and Tables

**Table 1:** PICOS eligibility criteria.

Criteria	Determinants
Population	People living with HIV
Intervention	Antiretroviral therapy
Comparison	There was no control, as this was a one-arm study.
Outcome	Depression/depressive symptoms
Design	Cross-sectional

**Table 2:** General overview characteristics of included cross-sectional studies.

Author	Sample Size	Age (Years) Mean ± SD	Gender (m/f)	Number with Depression	% with Depression	Study Design	Method of Measuring Depression	Country	Overall Quality
Shumba et al., 2013 [31]	732	NR	228/504	431	59.00	Cross-sectional	Adherence survey questionnaire that included a depressive symptoms score.	Uganda	High
Namagga et al., 2021 [39]	393	NR	105/288	106	27.00	Cross-sectional	Beck Depression Inventory Scale I (BDI- I)	Uganda	High
Dlulane et al., 2021 [32]	334	NR	104/230	145	44.00	Cross-sectional	Beck Depression Inventory (BDI-II)	South Africa	High
Bernard et al., 2020 [37]	334	NR	109/165	60	17.9	Cross-sectional	Centre for Epidemiological Studies Depression Scale (CES-D)	Cote D’Ivoire and Senegal	Moderate
Kitshoff et al., 2014 [42]	146	NR	40/106	91	62.00	Cross-sectional	CES-D	South Africa	Moderate
Hanass-Hancock et al., 2019 [46]	1042	38.1 ± NR	358/684	271	26.00	Cross-sectional	CES-D	South Africa	High
Yeneabat et al., 2017 [41]	390	35.78 ± 9.26	128/262	299	76.7	Cross-sectional	CES-D	Ethiopia	Moderate
Coppenhagen and Duvenage, 2019 [18]	479	38.9 ± 8.72	227/395	248	51.7	Cross-sectional	CES-D	South Africa	High
Yeji (a) et al., 2014 [27]	272	37 ± 8.6	58/214	90	33.00	Cross-sectional	General Health Questionnaire (GHQ12)	South Africa	Moderate
Yeji (b) et al., 2014 [27]	272	37 ± 8.6	58/214	98	36.00	Cross-sectional	C-GHQ	South Africa	Moderate
Yeji (c) et al., 2014 [27]	272	37 ± 8.6	58/214	103	38.00	Cross-sectional	Likert Method	South Africa	Moderate
Goehler et al., 2018 [20]	154	NR	84/73	33	21.4	Cross-sectional	Hopkins Symptom Checklist for Depression (HSCL)	Uganda	Moderate
Tareke et al., 2018 [48]	407	36.9 ± 10.5	173/234	198	48.6	Cross-sectional	Hospital Anxiety and Depression Scale (HADS) questionnaire	Ethiopia	High
Degu et al., 2023 [22]	404	36.75 ± NR	149/255	153	38.00	Cross-sectional	HADS questionnaire	Ethiopia	High
Camara et al., 2019 [47]	160	40.6 ± 10.8	42/118	14	51.90	Cross-sectional	HADS questionnaire	Republic of Guinea	Moderate
Malava et al., 2018 [23]	103	38 ± 10	37/66	9	9.00	Cross-sectional	Patient Health Questionnaire (PHQ-9)	Malawi	High
Parcesepe et al., 2021 [35]	8675	NR	4196/8280	2663	30.7	Cross-sectional	PHQ-9	Cameroon	High
Duko et al., 2018 [34]	401	38 ± 10.23	272/129	195	48.6	Cross-sectional	PHQ-9	Ethiopia	High
Desta et al., 2022 [25]	554	38 ± 9.86	277/277	249	44.9	Cross-sectional	PHQ-9	Ethiopia	High
Gebrezgiabher et al., 2019 [45]	411	36.1 ± 9.2	239/172	60	14.6	Cross-sectional	PHQ-9	Ethiopia	High
Mohamud et al., 2023 [44]	331	NR	162/169	111	33.5	Cross-sectional	PHQ-9	Somalia	High
Adedeji et al., 2023 [26]	172	44.3 ± 11.7	44/128	28	16.3	Cross-sectional	PHQ-9	Nigeria	Moderate
Adewole et al., 2021 [40]	279	43.1 ± 10.3	90/189	67	24.00	Cross-sectional	PHQ-9	Nigeria	High
Beyamo et al., 2020 [30]	410	33.05 ± 9.341	177/233	208	50.5	Cross-sectional	PHQ-9	Ethiopia	High
Gebru et al., 2024 [21]	420	42.8 ± 10.7	151/269	220	52.4	Cross-sectional	PHQ-9	Ethiopia	High
Abadiga et al., 2019 [33]	393	25.6 ± 9.45	177/216	164	41.7	Cross-sectional	PHQ-9	Ethiopia	High
Hankebo et al., 2023 [28]	392	42.8 ± 10.7	168/224	148	37.8	Cross-sectional	PHQ-9	Ethiopia	High
Seid et al., 2020 [36]	395	NR	153/242	79	20.00	Cross-sectional	PHQ-9	Ethiopia	High
Mekonen et al., 2021 [24]	530	38.85 ± 10.72	160/370	88	16.6	Cross-sectional	PHQ-9	Ethiopia	High
Madundo et al., 2023 [52]	272	41 ± 12	106/166	112	41.00	Cross-sectional	PHQ-9	Tanzania	High
Ngum et al., 2017 [43]	300	40.9 ± 9.7	80/220	80	26.7	Cross-sectional	PHQ-9	Cameroon	Moderate
Mandlate et al., 2023 [19]	395	36.7 ± 9.8	120/275	108	27.34	Cross-sectional	Structured diagnostic interview, the Brazilian version of the Portuguese language Mini InternationalNeuropsychiatric Interview (MINI) Plus 4.0.0	Mozambique	Moderate
Bongongo et al., 2013 [38]	117	NR	35/82	84	71.8	Cross-sectional	Zung Self-Rating Depression Scale	South Africa	Moderate
Vezi et al., 2024 [51]	121	NR	57/64	21	17.4	Cross-sectional	PHQ-9	South Africa	Moderate
Amha et al., 2022 [50]	266	NR	102/164	104	39.1	Cross-sectional	PHQ-9	Ethiopia	High
Nyogesa et al., 2029 [49]	450	42.7 ± 9.7	94/356	153	37.7	Cross-sectional	PHQ-9	Kenya	High

PLWHIV: people living with HIV; NR: not reported; ART: antiretroviral therapy; m: male; f: female; PHQ: Patient Health Questionnaire, HADS: Hospital Anxiety and Depression Scale, CES-D: Centre for Epidemiological Study Depression Scale, BDI-II: Beck Depression Inventory.

**Table 3:** Meta-regression based on region and methods of measuring depression.

Moderator	Estimate	SE	Z	*p*	CI Lower Bound	CI Upper Bound
African region	0.0116	0.0259	0.447	0.655	−0.039	0.062
Method of measuring depression	0.0173	0.0150	1.15	0.250	−0.012	0.047

SE: standard error; CI: confidence intervals.

## Supplementary Materials

The following supporting information can be downloaded at: https://www.mdpi.com/article/10.3390/healthcare13010085/s1, Supplementary File S1: PRISMA 2020 Checklist. Supplementary File S2 Table S1: Quality assessment of cross-sectional studies, according to the Newcastle–Ottawa Checklist. Supplementary File S2 Figure S1: Subgroup meta-analysis based on the method of measuring depression.
