# Supplementary material for: Prevalence of Depression Among People Living with HIV on Antiretroviral Therapy in Africa: A Systematic Review and Meta-Analysis
Source: Healthcare (Basel). 2025 Jan 6;13(1):85. doi: 10.3390/healthcare13010085 (PMC11719924; doi:10.3390/healthcare13010085)
Supplement: Supplementary file 1 [file healthcare-13-00085-s001.zip › Supplementary File S2.pdf]

Table S1: Quality assessment of Cross-sectional Studies according to New Castle Ottawa checklist

Authors	Selection				Comparability	Outcome		Score	Quality
	Representativeness of the sample	Sample size	Nonrespondents	Ascertainment of exposure	Based on design and analysis	Assessment of outcome	Statistical test		
Shumba et al., 2013 [1]	★	★	☆	★	★★	★	★	7	High
Namagga et al., 2021 [2]	★	★	☆	★	★★	★	★	7	High
Dlulane et al., 2021 [3]	★	★	☆	★	★★	★	★	7	High
Bernard et al., 2020 [4]	☆	★	☆	★	★★	★	★	6	Moderate
Kitshoff et al., 2014 [5]	☆	★	☆	★	★★	★	★	6	Moderate
Hanass-Hancock et al., 2019 [6]	★	★	☆	★	★★	★	★	7	High
Yeneabat et al., 2017 [7]	☆	★	★	★	★★	★	★	7	High
Coppenhagen and Duvenage 2019 [8]	★	★	☆	★	★★	★	★	7	High
Yeji (a) et al., 2014 [9]	☆	★	☆	★	★★	★	★	6	Moderate
Yeji (b) et al., 2014 [9]	☆	★	☆	★	★★	★	★	6	Moderate
Yeji (c) et al., 2014 [9]	☆	★	☆	★	★★	★	★	6	Moderate
Goehler et al., 2018 [10]	☆	★	☆	★	★★	★	★	6	Moderate
Tareke et al., 2018 [11]	★	★	☆	★	★★	★	★	7	High
Degu et al., 2023 [12]	★	★	☆	★	★★	★	★	7	High
Camara et al., 2019 [13]	★	★	★	★	★★	★	★	8	High
Malava et al., 2018 [14]	☆	★	☆	★	★★	★	★	6	Moderate
Parcesepe et al., 2021 [15]	★	★	☆	★	★★	★	★	7	High
Duko et al., 2018 [16]	☆	★	☆	★	★★	★	★	6	Moderate
Desta et al., 2022 [17]	★	★	☆	★	★★	★	★	7	High
Gebrezgiabher et al., 2019 [18]	★	★	★	★	★★	★	★	8	High
Mohamud et al., 2023 [19]	★	★	☆	★	★★	★	★	7	High

Adedeji et al., 2023 [20]	☆	★	☆	★	★★	★	★	6	Moderate
Adewole et al., 2021 [21]	★	★	☆	★	★★	★	★	7	High
Beyamo et al., 2020 [22]	★	★	☆	★	★★	★	★	7	High
Gebru et al., 2024 [23]	★	★	☆	★	★★	★	★	7	High
Abadiga et al., 2019 [24]	★	★	☆	★	★★	★	★	7	High
Hankebo et al., 2023 [25]	★	★	☆	★	★★	★	★	7	High
Seid et al., 2020 [26]	★	★	★	★	★★	★	★	8	High
Mekonen et al., 2021 [27]	★	★	★	★	★★	★	★	8	High
Madundo et al., 2023 [28]	★	★	☆	★	★★	★	★	7	High
Ngum et al., 2017 [29]	☆	★	☆	★	★★	★	★	6	Moderate
Mandlate et al., 2023 [30]	☆	★	☆	★	★★	★	★	6	Moderate
Bongongo et al., 2013 [31]	☆	★	☆	★	★★	★	★	6	Moderate
Vezi et al., 2024 [32]	☆	★	☆	★	★★	★	★	6	Moderate
Amha et al., 2022 [33]	★	★	★	★	★★	★	★	8	High
Nyogesa et al., 2019 [34]	☆	★	★	★	★★	★	★	7	High

Subgroup	Events	Total	Weight	IV, Random, 95% CI
Subgroup = 6				
Shumba et al., 2013	431	732	2.8%	0.59 [0.55; 0.62]
Yeji (c) et al., 2014	103	272	2.8%	0.38 [0.32; 0.44]
Goehler et al., 2018	33	154	2.7%	0.21 [0.15; 0.29]
Mandlate et al., 2023	108	395	2.8%	0.27 [0.23; 0.32]
Bongongo et al., 2013	84	117	2.7%	0.72 [0.63; 0.80]
Total (95% CI)		1670	13.8%	0.43 [0.25; 0.62]
Heterogeneity: $Tau^2 = 0.0440$; $Chi^2 = 217.7$, df = 4 ($P < 0.01$); $I^2 = 98\%$				
Subgroup = 4				
Namagga et al., 2021	106	393	2.8%	0.27 [0.23; 0.32]
Dlulane et al., 2021	145	334	2.8%	0.43 [0.38; 0.49]
Total (95% CI)		727	5.6%	0.35 [0.19; 0.51]
Heterogeneity: $Tau^2 = 0.0129$; $Chi^2 = 21.86$, df = 1 ($P < 0.01$); $I^2 = 95\%$				
Subgroup = 2				
Bernard et al., 2020	60	334	2.8%	0.18 [0.14; 0.23]
Kitshoff et al., 2014	91	146	2.7%	0.62 [0.54; 0.70]
Hanass-Hancock et al., 2019	271	1042	2.8%	0.26 [0.23; 0.29]
Yeneabat et al., 2017	299	390	2.8%	0.77 [0.72; 0.81]
Coppenhagen and Duvenage 2019	248	479	2.8%	0.52 [0.47; 0.56]
Total (95% CI)		2391	13.9%	0.47 [0.25; 0.68]
Heterogeneity: $Tau^2 = 0.0602$; $Chi^2 = 566.07$, df = 4 ($P < 0.01$); $I^2 = 99\%$				
Subgroup = 5				
Yeji (a) et al., 2014	90	272	2.8%	0.33 [0.28; 0.39]
Yeji (b) et al., 2014	98	272	2.8%	0.36 [0.30; 0.42]
Total (95% CI)		544	5.5%	0.35 [0.31; 0.39]
Heterogeneity: $Tau^2 = 0$; $Chi^2 = 0.52$, df = 1 ($P = 0.47$); $I^2 = 0\%$				
Subgroup = 3				
Tareke et al., 2018	198	407	2.8%	0.49 [0.44; 0.54]
Degu et al., 2023	153	404	2.8%	0.38 [0.33; 0.43]
Camara et al., 2019	14	160	2.8%	0.09 [0.05; 0.14]
Total (95% CI)		971	8.4%	0.32 [0.08; 0.55]
Heterogeneity: $Tau^2 = 0.0421$; $Chi^2 = 157.75$, df = 2 ($P < 0.01$); $I^2 = 99\%$				
Subgroup = 1				
Malava et al., 2018	9	103	2.8%	0.09 [0.04; 0.16]
Parcesepe et al., 2021	2663	8675	2.8%	0.31 [0.30; 0.32]
Duko et al., 2018	195	401	2.8%	0.49 [0.44; 0.54]
Desta et al., 2022	249	554	2.8%	0.45 [0.41; 0.49]
Gebrezgiabher et al., 2019	60	411	2.8%	0.15 [0.11; 0.18]
Mohamud et al., 2023	111	331	2.8%	0.34 [0.28; 0.39]
Adedeji et al., 2023	28	172	2.8%	0.16 [0.11; 0.23]
Adewole et al., 2021	67	279	2.8%	0.24 [0.19; 0.29]
Beyamo et al., 2020	208	410	2.8%	0.51 [0.46; 0.56]
Gebru et al., 2024	220	420	2.8%	0.52 [0.47; 0.57]
Abadiga et al., 2019	164	393	2.8%	0.42 [0.37; 0.47]
Hankebo et al., 2023	148	392	2.8%	0.38 [0.33; 0.43]
Seid et al., 2020	79	395	2.8%	0.20 [0.16; 0.24]
Mekonen et al., 2021	88	530	2.8%	0.17 [0.14; 0.20]
Madundo et al., 2023	112	272	2.8%	0.41 [0.35; 0.47]
Ngum et al., 2017	80	300	2.8%	0.27 [0.22; 0.32]
Vezi et al., 2024	21	121	2.7%	0.17 [0.11; 0.25]
Amha et al., 2022	104	266	2.8%	0.39 [0.33; 0.45]
Nyogesa et al., 2019	153	450	2.8%	0.34 [0.30; 0.39]
Total (95% CI)		14875	52.9%	0.32 [0.25; 0.38]
Heterogeneity: $Tau^2 = 0.0174$; $Chi^2 = 595.88$, df = 18 ($P < 0.01$); $I^2 = 97\%$				
**Total (95% CI)**		**21178**	**100.0%**	**0.36 [0.30; 0.41]**

Heterogeneity: $Tau^2 = 0.0271$; $Chi^2 = 1751.59$, df = 35 ($P = 0$); $I^2 = 98\%$

Test for subgroup differences: [illegible]

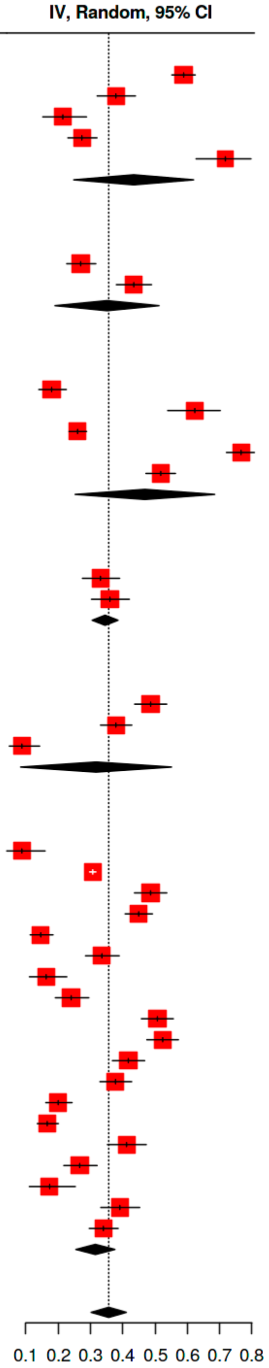

Figure S1: Subgroup meta-analysis based on the method of measuring depression. Shumba et al., 2013 [1], Namagga et al., 2021 [2], Dlulane et al., 2021 [3], Bernard et al., 2020 [4], Kitshoff et al., 2014 [5], Hanass-Hancock et al., 2019 [6], Yeneabat et al., 2017 [7], Coppenhagen and Duvenage 2019 [8], Yeji (a) et al., 2014 [9], Yeji (b) et al., 2014 [9], Yeji (c) et al., 2014 [9], Goehler et al., 2018 [10], Tareke et al., 2018 [11], Degu et al., 2023 [12], Camara et al., 2019 [13], Malava et al., 2018 [14], Parcesepe et al., 2021 [15], Duko et al., 2018 [16], Desta et al., 2022 [17], Gebrezgiabher et al., 2019 [18], Mohamud et al., 2023 [19], Adedeji et al., 2023 [20], Adewole et al., 2021 [21], Beyamo et al., 2020 [22], Gebru et al., 2024 [23], Abadiga et al., 2019 [24], Hankebo et al., 2023 [25], Seid et al., 2020 [26], Mekonen et al., 2021 [27], Madundo et al., 2023 [28], Ngum et al., 2017 [29], Mandlate et al., 2023 [30], Bongongo et al., 2013 [31],Vezi et al., 2024 [32], Amha et al., 2022 [33], Nyogesa et al., 2029 [34].
Subgroup 1, used patient health questionnaire (PHQ-9) method; subgroup 2, used epidemiological studies depression scale (CES-D), subgroup 3, used hospital anxiety and depression (HAD) scale questionnaire, subgroup 4, used Beck depression inventory scale, class 5, one study used general health questionnaire (GHQ12)

and subgroup 6, was subgrouped into other, these used different methods (Adherence survey questionnaire that included a depressive symptoms score, Likert method, Hopkins symptom checklist for depression (HSCL), structured diagnostic interview, the Brazilian version of the Portuguese language mini-international neuropsychiatric interview (MINI) Plus 4.0.0 and Zung self-rating depression scale).

References

[1] Shumba C, Atukunda R, Imakit R, Memiah P. Prevalence of depressive symptoms amongst highly active antiretroviral therapy (HAART) patients in AIDSrelief Uganda. J Public Health Afr 2013;4:84–7. https://doi.org/10.4081/jphia.2013.e19.

[2] Namagga JK, Rukundo GZ, Niyonzima V, Voss J. Depression and HIV associated neurocognitive disorders among HIV infected adults in rural southwestern Uganda: a cross-sectional quantitative study. BMC Psychiatry 2021;21. https://doi.org/10.1186/s12888-021-03316-w.

[3] Dlulane YZ, Apalata T, Abaver DT. Prevalence of depression among HIV positive adults on antiretroviral therapy in O.R. Tambo District, Eastern Cape, South Africa: A descriptive cross-sectional study. From: African Journal for Physical Activity and Health Sciences (AJPHES) 2021;27. https://doi.org/10.37597/ajphes.2021.27A5.

[4] Bernard C, Font H, Diallo Z, Ahonon R, Tine JM, N'Guessan Abouo F, et al. Prevalence and factors associated with severe depressive symptoms in older west African people living with HIV. BMC Psychiatry 2020;20. https://doi.org/10.1186/s12888-020-02837-0.

[5] Kitshoff C, Campbell L, Naidoo SS. The association between depression and adherence to antiretroviral therapy in HIV-positive patients, KwaZulu-Natal, South Africa. South African Family Practice 2012;54:145–50. https://doi.org/10.1080/20786204.2012.10874194.

[6] Hanass-Hancock J, Carpenter B, Myezwa H. The missing link: exploring the intersection of gender, capabilities, and depressive symptoms in the context of chronic HIV. Women Health 2019;59:1212–26. https://doi.org/10.1080/03630242.2019.1607799.

[7] Yeneabat T, Bedaso A, Amare T. Factors associated with depressive symptoms in people living with HIV attending antiretroviral clinic at Fitche Zonal Hospital, Central Ethiopia: Cross-sectional study conducted in 2012. Neuropsychiatr Dis Treat 2017;13:2125–31. https://doi.org/10.2147/NDT.S131722.

[8] Van Coppenhagen B, Duvenage HS. South African Journal of Psychiatry 2019. https://doi.org/10.4102/sajpsychiatry.

[9] Yeji F, Klipstein-Grobusch K, Newell ML, Hirschhorn LR, Hosegood V, Bärnighausen T. Are social support and HIV coping strategies associated with lower depression in adults on antiretroviral treatment? Evidence from rural KwaZulu-Natal, South Africa. AIDS Care - Psychological and Socio-Medical Aspects of AIDS/HIV 2014;26:1482–9. https://doi.org/10.1080/09540121.2014.931561.

[10] Manne-Goehler J, Kakuhikire B, Abaasabyoona S, Bärnighausen TW, Okello S, Tsai AC, et al. Depressive Symptoms Before and After Antiretroviral Therapy Initiation Among Older-Aged Individuals in Rural Uganda. AIDS Behav 2019;23:564–71. https://doi.org/10.1007/s10461-018-2273-4.

[11] Tareke M, Addisu F, Abate A. Depression among patients attending antiretroviral treatment program in public health facilities in Bahir Dar City, Ethiopia. J Affect Disord 2018;232:370–4. https://doi.org/10.1016/j.jad.2018.02.078.

[12] Degu FS. Anxiety and Depression Disorder among Adult People Living with HIV/AIDS on Follow-up at Dessie Public Health Facilities Antiretroviral Therapy Clinics, Northeast Ethiopia: A Multicenter Cross-sectional Study. Open AIDS J 2023;17. https://doi.org/10.2174/0118746136250239231025074541.

[13] Camara A, Sow MS, Touré A, Sako FB, Camara I, Soumaoro K, et al. Anxiety and depression among HIV patients of the infectious disease department of Conakry University Hospital in 2018. Epidemiol Infect 2020;148. https://doi.org/10.1017/S095026881900222X.

[14] Malava JK, Lancaster KE, Hosseinipour MC, Rosenberg NE, O'donnell JK, Kauye F, et al. Prevalence and correlates of probable depression diagnosis and suicidal ideation among patients receiving HIV care in Lilongwe, Malawi. Malawi Medical Journal 2018;30:236–42. https://doi.org/10.4314/mmj.v30i4.5.

[15] Parcesepe AM, Remch M, Dzudie A, Ajeh R, Nash D, Anastos K, et al. Depressive Symptoms, Gender, Disclosure, and HIV Care Stage Among People Living with HIV in Cameroon. AIDS Behav 2022;26:651–61. https://doi.org/10.1007/s10461-021-03425-3.

[16] Duko B, Geja E, Zewude M, Mekonen S. Prevalence and associated factors of depression among patients with HIV/AIDS in Hawassa, Ethiopia, cross-sectional study. Ann Gen Psychiatry 2018;17. https://doi.org/10.1186/s12991-018-0215-1.

[17] Desta F, Tasew A, Tekalegn Y, Zenbaba D, Sahiledengle B, Assefa T, et al. Prevalence of depression and associated factors among people living with HIV/AIDS in public hospitals of Southeast Ethiopia. BMC Psychiatry 2022;22. https://doi.org/10.1186/s12888-022-04205-6.

[18] Beyene Gebrezgiabher B, Huluf Abraha T, Hailu E, Siyum H, Mebrahtu G, Gidey B, et al. Depression among Adult HIV/AIDS Patients Attending ART Clinics at Aksum Town, Aksum, Ethiopia: A Cross-Sectional Study. Depress Res Treat 2019;2019. https://doi.org/10.1155/2019/3250431.
[19] Mohamud AK, Ahmed OA, Mohamud AA, Dirie NI. Prevalence of and factors associated with depression among adult patients living with HIV/AIDs undergoing ART unit in Banadir hospital, Mogadishu Somalia. BMC Psychiatry 2023;23. https://doi.org/10.1186/s12888-023-04723-x.
[20] Adedeji WA, Ma Q, Raji AM, Cha R, Rasaki OM, Hutson A, et al. Prevalence of depression among people living with HIV in rural hospitals in South-Western Nigeria-Association with clinico-demographic factors. AIDS Res Ther 2023;20. https://doi.org/10.1186/s12981-023-00586-0.
[21] Adewole OE, Olagundoye OA, Ajumobi IO. Depression and its associated factors among people living with hiv/aids attending the hiv/aids clinic in southwest nigeria. Family Medicine and Primary Care Review 2021;23:7–12. https://doi.org/10.5114/fmpcr.2021.103149.
[22] Beyamo A, Bashe T, Facha W, Moshago T. Depression and associated factors among adult hiv/aids patients attending antiretroviral therapy at wolaita sodo university teaching and referral hospital, Southern Ethiopia. HIV/AIDS - Research and Palliative Care 2020;12:707–15. https://doi.org/10.2147/HIV.S278794.
[23] Gebru T, Ejara D, Yalew A, Deyessa N. Prevalence of depression and associated factors among HIV/AIDS patients attending antiretroviral therapy clinic at Adama Hospital Medical College, Adama, Central Ethiopia. Sci Rep 2024;14. https://doi.org/10.1038/s41598-024-52142-z.
[24] Abadiga M. Depression and its associated factors among HIV/AIDS patients attending ART clinics at Gimbi General hospital, West Ethiopia, 2018. BMC Res Notes 2019;12. https://doi.org/10.1186/s13104-019-4553-0.
[25] Hankebo M, Fikru C, Lemma L, Aregago G. Depression and Associated Factors among People Living with Human Immunodeficiency Virus Attending Antiretroviral Therapy in Public Health Facilities, Hosanna Town, Southern Ethiopia. Depress Res Treat 2023;2023. https://doi.org/10.1155/2023/7665247.
[26] Seid S, Abdu O, Mitiku M, Tamirat KS. Prevalence of depression and associated factors among HIV/AIDS patients attending antiretroviral therapy clinic at Dessie referral hospital, South Wollo, Ethiopia. Int J Ment Health Syst 2020;14. https://doi.org/10.1186/s13033-020-00389-0.
[27] Mekonen T, Belete H, Fekadu W. Depressive symptoms among people with HIV/AIDS in Northwest Ethiopia: Comparative study. BMJ Open 2021;11. https://doi.org/10.1136/bmjopen-2021-048931.
[28] Madundo, K.; Knettel, B.A.; Knippler, E.; Mbwambo, J. Prevalence, Severity, and Associated Factors of Depression in Newly Diagnosed People Living with HIV in Kilimanjaro, Tanzania: A Cross-Sectional Study. BMC Psychiatry 2023, 23, 83. https://doi.org/10.1186/s12888-022-04496-9.
[29] Ngum PA, Fon PN, Ngu RC, Verla VS, Luma HN. Depression Among HIV/AIDS Patients on Highly Active Antiretroviral Therapy in the Southwest Regional Hospitals of Cameroon: A Cross-Sectional Study. Neurol Ther 2017;6:103–14. https://doi.org/10.1007/s40120-017-0065-9.
[30] Mandlate FM, Greene MC, Pereira LF, Gouveia ML, Mari JJ, Cournos F, et al. Association between mental disorders and adherence to antiretroviral treatment in health facilities in two Mozambican provinces in 2018: a cross-sectional study. BMC Psychiatry 2023;23. https://doi.org/10.1186/s12888-023-04782-0.
[31] Bongongo T, Tumbo J, Govender I. Depressive features among adult patients receiving antiretroviral therapy for HIV in Rustenburg district, SA. South African Journal of Psychiatry 2013;19:31–4. https://doi.org/10.7196/SAJP.418.
[32] Vezi ZL, Rangiah S, Naidoo K. South African Family Practice. S Afr Fam Pract 2024;66:5821. https://doi.org/10.4102/safp.
[33] Amha H, Denekew B, Asnakew S. Depressive symptoms and associated factors among adults attending antiretroviral therapy clinic in Debre Markos comprehensive specialized hospital, Amhara, Ethiopia. SAGE Open Med 2022;10. https://doi.org/10.1177/20503121221100992.
[34] Nyongesa MK, Mwangi P, Wanjala SW, Mutua AM, Newton CRJC, Abubakar A. Prevalence and correlates of depressive symptoms among adults living with HIV in rural Kilifi, Kenya. BMC Psychiatry 2019;19. https://doi.org/10.1186/s12888-019-2339-5.
